# Long-Term Renal Outcomes Following Left Renal Vein Ligation Versus Direct Splenorenal Shunt Ligation in Living Donor Liver Transplantation: A 10-Year Single-Center Study

**DOI:** 10.3389/ti.2026.16021

**Published:** 2026-02-18

**Authors:** Woo-Hyoung Kang, Deok-Bog Moon, Shin Hwang, Ki-Hun Kim, Chul-Soo Ahn, Tae-Yong Ha, Gi-Won Song, Dong-Hwan Jung, Gil-Chun Park, Young-In Yoon, Byeong-Gon Na, Sang-Hoon Kim, Sung-Min Kim, Sung-Gyu Lee

**Affiliations:** Division of Liver Transplantation and Hepatobiliary Surgery, Department of Surgery, Asan Medical Center, University of Ulsan College of Medicine, Seoul, Republic of Korea

**Keywords:** end-stage liver disease, left renal vein ligation, living donor liver transplantation, nephrotoxicity, splenorenal shunt ligation

## Abstract

In living donor liver transplantation (LDLT), large splenorenal shunts (SRS) can divert portal inflow and negatively affect graft function due to portal steal syndrome. Direct SRS ligation (SRSL) and left renal vein ligation (LRVL) are used to prevent this complication; however, the long-term renal impact of LRVL remains unclear, particularly in recipients requiring nephrotoxic immunosuppression. We retrospectively analyzed adult LDLT recipients with large SRS (>1 cm) and normal baseline renal function who underwent SRSL (n = 120) or LRVL (n = 74). Patient and graft survival, serial renal function profiles, and tacrolimus trough levels were evaluated. Survival outcomes were comparable between the two groups. LRVL was more frequently performed in patients with higher preoperative Model for End-Stage Liver Disease (MELD) scores or increased transfusion requirements. During long-term follow-up, the LRVL group showed a more evident decline in renal function, with persistently higher serum creatinine levels, despite similar tacrolimus exposure. Four recipients in the LRVL group progressed to end-stage renal disease requiring dialysis within 10 years, whereas no dialysis cases occurred following SRSL. Although both strategies are clinically feasible, LRVL demonstrated a stronger association with progressive renal deterioration. These findings suggest that SRSL may be preferred in recipients with renal vulnerability to minimize cumulative renal burden.

## Graphical Abstract

**Figure:**
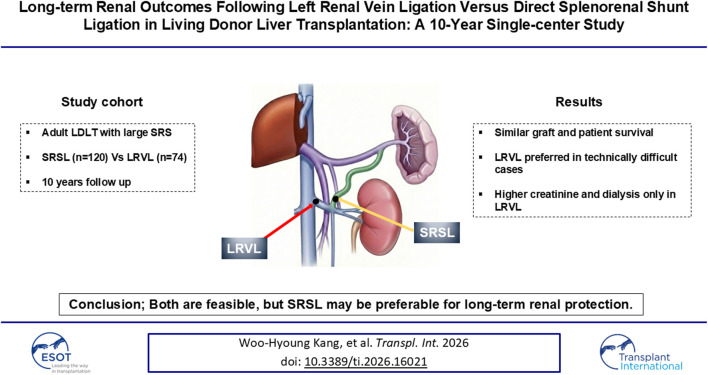

## Introduction

In patients with end-stage liver disease, portal hypertension frequently leads to the formation of spontaneous portosystemic shunts, including coronary, periumbilical, and splenorenal shunts (SRS). Among these, large SRSs can divert portal blood flow away from the liver graft after living donor liver transplantation (LDLT), resulting in portal steal syndrome. This condition may compromise graft regeneration and function, potentially leading to graft failure. Therefore, intraoperative interruption of large shunts is essential to ensure adequate portal inflow and graft survival [1–3].

The following two major surgical approaches are used to interrupt SRSs: Direct splenorenal shunt ligation (SRSL) and left renal vein ligation (LRVL) [4, 5]. SRSL involves anatomically precise identification and ligation of the shunt vessel itself, but often present technical challenges during the procedure (particularly in patients with severe adhesions or complex vascular anatomy) and carries a risk of bleeding from surrounding tissues or from the shunt itself. In contrast, LRVL achieves functional interruption of the SRS by ligating the left renal vein (LRV), and is often preferred due to its simplicity and ease of access.

Our institution previously reported the safety and feasibility of LRVL in LDLT, and several subsequent studies have confirmed its effectiveness in preventing portal steal syndrome and maintaining stable portal perfusion [1, 4]. However, concerns persist regarding the potential impact of LRVL on renal function. Because the LRV is the major venous drainage pathway for the left kidney, its ligation may lead to venous congestion and impaired renal perfusion. Although previous nontransplant studies have suggested that LRV ligation has minimal long-term effects on renal function—because collateral pathways such as the left gonadal vein can provide sufficient outflow—the applicability of these findings to transplant recipients remains to be evaluated [6–9].

To date, most studies on LRVL in LDLT have focused on surgical feasibility and short-term safety, with few addressing long-term renal outcomes. Moreover, there is a lack of direct comparisons between SRSL and LRVL in the transplant setting, especially using methodologies that adjust for baseline differences in renal function and comorbidities.

We aimed to compare the long-term outcomes of patients undergoing either LRVL or SRSL during LDLT, focusing on graft-related endpoints such as shunt recanalization, graft failure, and patient survival, as well as changes in renal function. To account for baseline differences, propensity score matching was applied, and renal function was assessed over a 10-year follow-up period in a single-center setting using a consistent immunosuppressive protocol.

## Materials and Methods

### Study Design and Patient Selection

This retrospective single-center cohort study was conducted at Asan Medical Center and included adult patients who underwent LDLT between January 2009 and December 2015. Among 267 patients with spontaneous SRSs >1 cm in diameter and preserved renal function (serum creatinine ≤1.4 mg/dL), 194 were selected for analysis based on having undergone one of the two intraoperative shunt interruption techniques: LRVL (n = 74) or SRSL (n = 120). The remaining 73 patients were excluded because they had received alternative treatments for SRS—such as selective embolization, plug-assisted retrograde transvenous obliteration, or proximal splenic vein embolization [5]—or were managed with immunosuppressive regimens other than tacrolimus.

### Surgical Techniques

The method for SRS interruption was determined intraoperatively based on a comprehensive assessment of anatomical feasibility, severity of adhesions, complexity of collateral circulation, and hemodynamic stability. Portal hemodynamics were primarily evaluated using real-time cine-portography. Portal pressure was not routinely measured. The presence of portal flow diversion through collateral vessels, as well as the velocity and direction of portal flow, was directly visualized on cine-portography. Restoration of hepatopetal flow after ligation was confirmed jointly by the transplant surgeon and the interventional radiologist. In all cases, intraoperative portography was performed to verify adequate portal inflow and confirm the absence of residual portal steal [10].

Dissection of the SRS or the LRV was carried out either during total hepatectomy or after graft implantation. However, ligation was typically performed after portal reperfusion to avoid portal hypertension and ensure adequate graft inflow.

**FIGURE 1:**
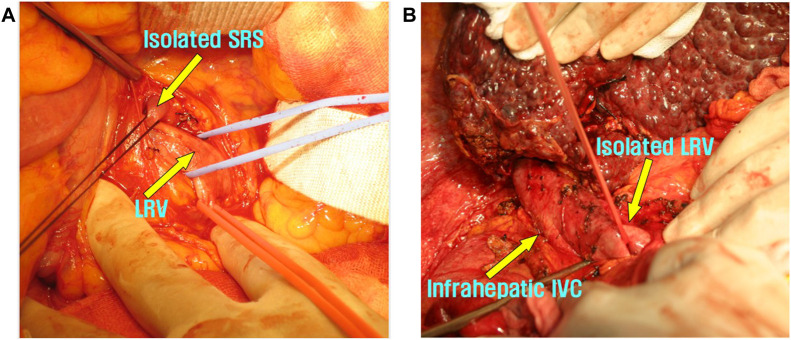
Representative illustrations of the available techniques to prevent splenorenal shunt (SRS). **(A)** Isolation of the splenorenal shunt for direct ligation (SRSL). **(B)** Isolation of the left renal vein after Kocherization for left renal vein ligation (LRVL).

In the SRSL group, patients with a single collateral draining into the LRV underwent direct ligation of the shunt. The mesocolon was dissected to expose the LRV near the ligament of Treitz or the inferior mesenteric vein. The shunt was then isolated circumferentially and ligated using thick silk ties or vascular nylon tape (Figure 1A). In the LRVL group, Kocherization was performed to mobilize the duodenum and mesocolon from the inferior vena cava, thereby exposing the LRV along its left side. After careful dissection, the LRV was completely encircled and fully ligated (Figure 1B). Compared with SRSL, this approach was technically simpler and provided a safer access route with lower risk of injury to adjacent vessels.

### Study Objectives and Endpoints

This study had two predefined objectives. The primary objective was to compare the long-term clinical outcomes of the two surgical approaches, including patient mortality, graft failure or need for retransplantation, and shunt recannulation. The secondary objective was to evaluate the differential effects of SRSL and LRVL on long-term renal function following a consistent immunosuppressive regimen.

### Renal Function and Immunosuppression Assessment

All patients had normal preoperative renal function and no history of kidney disease. Renal function was assessed using serum creatinine levels at postoperative intervals of 1 week, 1 month, 6 months, and 1, 3, 5, and 10 years. We also documented whether any patients required initiation of dialysis during the follow-up period. Patients in both groups received tacrolimus-based immunosuppression, and tacrolimus trough levels were analyzed as a supportive indicator to ensure comparable immunosuppressive exposure between the two groups. When tacrolimus dose reduction was required because of nephrotoxicity, mycophenolate mofetil or other non-nephrotoxic agents were added according to standard institutional protocols to maintain adequate immunosuppression.

### Statistical Analysis

To minimize baseline imbalances between the two groups and control for potential confounders affecting renal function, propensity score matching was performed. Matched variables included age, sex, graft-to-recipient weight ratio, Model for End-Stage Liver Disease (MELD) score, presence of diabetes mellitus and hypertension, preoperative serum creatinine, and intraoperative red blood cell transfusion volume [11]. One-to-one nearest-neighbor matching with a caliper of 0.2 was applied. After matching, balance between the groups was assessed using standardized mean differences. Because procedural selection was influenced by intraoperative findings and surgeon judgment, we included MELD score and intraoperative RBC transfusion volume as pragmatic surrogate indicators of overall surgical complexity and physiological instability. Although prior abdominal surgery and previous episodes of spontaneous bacterial peritonitis may partially reflect surgical difficulty, their impact is variable and difficult to quantify objectively. Surgeon-related factors could not be quantified and therefore could not be directly adjusted for.

For the longitudinal assessment of renal function, both fixed-effects models and linear mixed-effects models were used to account for repeated measurements over time. The postoperative follow-up period was divided into an early (0–1 years), an intermediate (1–5 years), and a late (5–10 years) phase, and temporal changes in creatinine levels as well as group-by-time interactions were evaluated accordingly. Period-specific changes in creatinine levels (ΔCr) were also analyzed to compare long-term renal trajectories between the LRVL and SRSL groups. Tacrolimus trough levels were analyzed using similar phase-specific fixed-effects models to evaluate differences in immunosuppression tapering patterns between the groups. Patient and graft survival were analyzed using the Kaplan–Meier method, and between-group differences were assessed with the log-rank test. Continuous variables were compared using Student’s t-test or the Mann–Whitney U test, while categorical variables were analyzed using the chi-square test or Fisher’s exact test, as appropriate. All statistical analyses were performed under the guidance of a medical statistician from our institution to ensure appropriate model selection and methodological validity.

This study was conducted with approval from the Institutional Review Board of Asan Medical Center (IRB No. AMC 2022-0763).

## Results

### Baseline Characteristics of the Original Cohort

A total of 194 adult LDLT recipients with large spontaneous SRSs were included in the analysis, comprising 120 patients in the SRSL group and 74 in the LRVL group. Most baseline characteristics were comparable between the two groups. The mean age was similar (52.3 vs. 53.0 years, p = 0.500), and the sex distribution was nearly identical (male participants: 67.5% vs. 67.6%). The presence of diabetes mellitus and hypertension, the primary liver disease patterns, the graft-to-recipient weight ratio, and the preoperative serum creatinine levels were also well balanced (Table 1).

**TABLE 1:** Baseline cohort characteristics.

Characteristic	Category	SRS ligation	LRV ligation	p-value	SMD
		120	74		
Sex (%)	Male	81 (67.5)	50 (67.6)	1.000	0.001
​	Female	39 (32.5)	24 (32.4)	​	​
Age (mean [SD])	​	52.33 (6.96)	53.00 (6.19)	0.500	0.101
Original disease (%)	HBV	83 (69.2)	51 (68.9)	0.661	0.287
​	HCV	6 (5.0)	2 (2.7)	​	​
​	ALD	17 (14.2)	11 (14.9)	​	​
​	PSC	1 (0.8)	0 (0.0)	​	​
​	AI	2 (1.7)	0 (0.0)	​	​
​	Other	11 (9.2)	10 (13.5)	​	​
GRWR (mean [SD])	​	1.17 (0.28)	1.14 (0.25)	0.437	0.117
MELD (mean [SD])	​	14.21 (6.27)	17.73 (5.98)	<0.001	0.575
DM (%)	No	93 (77.5)	52 (70.3)	0.339	0.165
​	Yes	27 (22.5)	22 (29.7)	​	​
HTN (%)	No	104 (86.7)	64 (86.5)	1.000	0.005
​	Yes	16 (13.3)	10 (13.5)	​	​
Preoperative serum creatinine (mean [SD])	​	0.72 (0.23)	0.72 (0.20)	0.882	0.022
Intraoperative RBC transfusion, unit (mean [SD])	​	7.85 (7.06)	15.36 (10.66)	<0.001	0.831

HBV, hepatitis B virus; HCV, hepatitis C virus; ALD, alcoholic liver disease; PSC, primary sclerosing cholangitis; AI, autoimmune hepatitis; GRWR, graft-to-recipient weight ratio; MELD, model for end-stage liver disease; DM, diabetes mellitus; HTN, hypertension; Cr, creatinine; RBC, red blood cell; SMD, standardized mean difference; SRS, splenorenal shunt; LRV, left renal vein.

Two variables demonstrated significant differences: The LRVL group exhibited a higher mean MELD score (17.7 ± 6.0 vs. 14.2 ± 6.3, p < 0.001), suggesting increased disease severity at transplantation. Additionally, intraoperative red blood cell transfusion volumes were noticeably higher in the LRVL group (15.4 ± 10.7 vs. 7.9 ± 7.1 units, p < 0.001). These findings suggest that LRVL was more frequently selected in surgically complex or hemodynamically challenging situations, rather than being applied uniformly across the cohort.

### Baseline Characteristics After Propensity Score Matching

To reduce potential confounding from preoperative or intraoperative variables that may influence long-term renal outcomes, 1:1 propensity score matching was performed using age, sex, MELD score, graft-to-recipient weight ratio, presence of diabetes and hypertension, preoperative serum creatinine levels, and intraoperative RBC transfusion volume. Matching with a caliper of 0.2 yielded 58 well-balanced pairs (n = 116).

After matching, most covariates achieved acceptable balance. Although minor residual imbalance remained in baseline creatinine, this difference was limited and clinically marginal. Overall, the matching process substantially improved comparability between the two groups (Table 2).

**TABLE 2:** Baseline characteristics in matched cohort.

Characteristic	Category	SRS ligation	LRV ligation	p-value	SMD
		58	58		
Sex (%)	Male	41 (70.7)	41 (70.7)	1	<0.001
​	Female	17 (29.3)	17 (29.3)	​	​
Age (mean [SD])	​	53.48 (7.68)	53.47 (5.32)	0.989	0.003
Original disease (%)	HBV	32 (55.2)	45 (77.6)	0.129	0.512
​	HCV	4 (6.9)	2 (3.4)	​	​
​	ALD	13 (22.4)	6 (10.3)	​	​
​	PSC	-	-	​	​
​	AI	1 (1.7)	0 (0.0)	​	​
​	Other	8 (13.8)	5 (8.6)	​	​
GRWR (mean [SD])	​	1.15 (0.29)	1.14 (0.26)	0.843	0.037
MELD (mean [SD])	​	16.93 (7.42)	16.33 (5.13)	0.612	0.095
DM (%)	No	40 (69.0)	39 (67.2)	1	0.037
​	Yes	18 (31.0)	19 (32.8)	​	​
HTN (%)	No	51 (87.9)	51 (87.9)	1	<0.001
​	Yes	7 (12.1)	7 (12.1)	​	​
Preoperative serum creatinine (mean [SD])	​	0.74 (0.25)	0.71 (0.19)	0.558	0.109
Intraoperative RBC transfusion, unit (mean [SD])	​	11.45 (8.07)	11.59 (8.22)	0.928	0.017

HBV, hepatitis B virus; HCV, hepatitis C virus; ALD, alcoholic liver disease; PSC, primary sclerosing cholangitis; AI, autoimmune hepatitis; GRWR, graft-to-recipient weight ratio; MELD, Model For End-Stage Liver Disease; DM, diabetes mellitus; HTN, hypertension; Cr, creatinine; RBC, red blood cell; SMD, standardized mean difference; SRS, splenorenal shunt; LRV, left renal vein.

### Clinical Outcomes in the Original and the Matched Cohorts

#### Patient and Graft Survival

**FIGURE 2:**
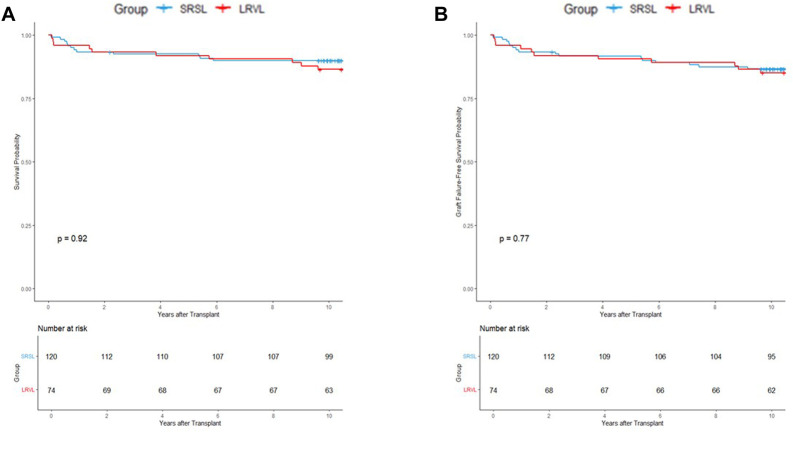
Kaplan–Meier survival curves for the overall cohort showing **(A)** patient survival and **(B)** graft survival. No significant differences were observed between the splenorenal shunt ligation (SRSL) and left renal vein ligation (LRVL) groups (p = 0.92 and 0.77, respectively).

In the original cohort (n = 194), Kaplan–Meier analysis demonstrated no significant difference in patient survival between the SRSL and LRVL groups (p = 0.92; Figure 2A). Similarly, graft failure–free survival did not differ significantly (p = 0.77; Figure 2B).

**FIGURE 3:**
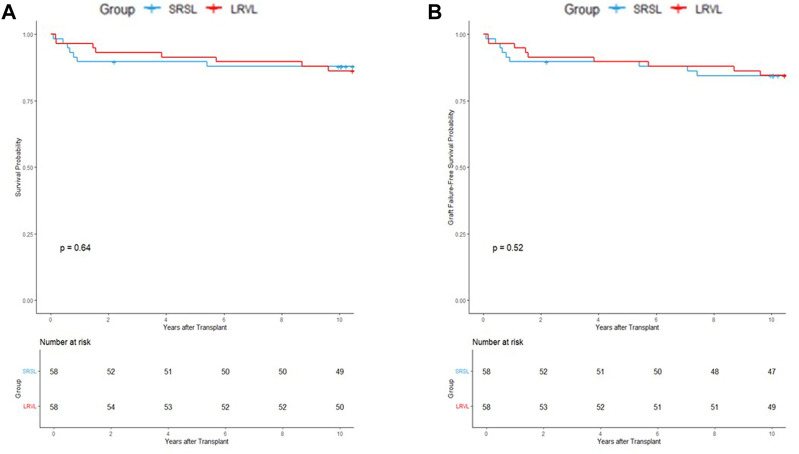
Kaplan–Meier survival curves for the propensity score–matched cohort showing **(A)** patient survival and **(B)** graft survival. No significant differences were observed between the splenorenal shunt ligation (SRSL) and left renal vein ligation (LRVL) groups (p = 0.64 and 0.52, respectively).

The findings were consistent with those from the propensity-matched cohort (n = 116). Neither patient survival (p = 0.64) nor graft survival (p = 0.52) showed intergroup differences (Figures 3A,B). These results indicate that both SRS interruption techniques offer comparable long-term survival outcomes.

#### Post-Transplant Dialysis Requirement

In the original cohort, four patients required chronic dialysis within the 10-year follow-up period, with all cases occurring in the LRVL group (4/74, 5.4%). Notably, each of these patients developed end-stage renal failure during the late postoperative period, between 5 and 10 years after transplantation. This pattern was consistent with that observed in the propensity score–matched cohort, in which all three cases requiring dialysis were likewise confined to the LRVL group, with none observed in the SRSL group.

Beyond the initial 10-year follow-up period, two additional patients in the LRVL group subsequently initiated dialysis at 11 and 14 years after transplantation, respectively (see Table 3). This suggests that the cumulative number of patients requiring dialysis is likely to increase further with a longer follow-up period. Taken together, these findings indicate that LRVL may be associated with a late-onset decline in renal function, underscoring the need for long-term renal surveillance in this population.

**TABLE 3:** Clinical characteristics of patients who progressed to dialysis after modulation of preoperative splenorenal shunt.

Patient No.	SRS Modulation	Sex	Etiology	Age (yr)	GRWR	MELD	Intraoperative RBC Transfusion (Units)	Pre-op Cr (mg/dL)	DM	HTN	Time to Dialysis (months)	Survival Status	Remarks
1	LRVL	M	HBV	44	1.05	14	16	0.8	Yes	No	83	Alive	—
2	LRVL	M	HBV	47	0.93	9	1	0.6	Yes	No	115	Alive	—
3	LRVL	M	HBV	47	1.08	24	80	0.7	No	No	95	Dead	Death due to pneumonia
4	LRVL	M	HBV	52	1.50	22	33	0.8	Yes	Yes	167	Alive	Dialysis initiated beyond 10-year follow-up
5	LRVL	M	HCV	41	0.94	21	59	0.6	No	No	95	Dead	Death due to post-retransplant bleeding
6	LRVL	M	HBV	47	1.38	31	15	0.6	Yes	Yes	137	Alive	Dialysis initiated beyond 10-year follow-up

LRVL, left renal vein ligation; HBV, hepatitis B virus; HCV, hepatitis C virus; GRWR, graft-to-recipient weight ratio; MELD, Model for End-Stage Liver Disease; RBC, red blood cell; Cr, creatinine; DM, diabetes mellitus; HTN, hypertension; SRS, splenorenal shunt.

#### Shunt Recanalization

Clinically significant shunt recanalization occurred only in the SRSL group (2/120, 1.7%). One case was detected at 1 month and the other at 10 years post-transplantation. Both were managed successfully using plug-assisted retrograde transvenous obliteration, with preserved graft function and stable renal parameters during subsequent follow-up. No recanalization events were identified in the LRVL group.

### Long-Term Renal Function Analysis

#### Temporal Trends in Serum Creatinine

**FIGURE 4:**
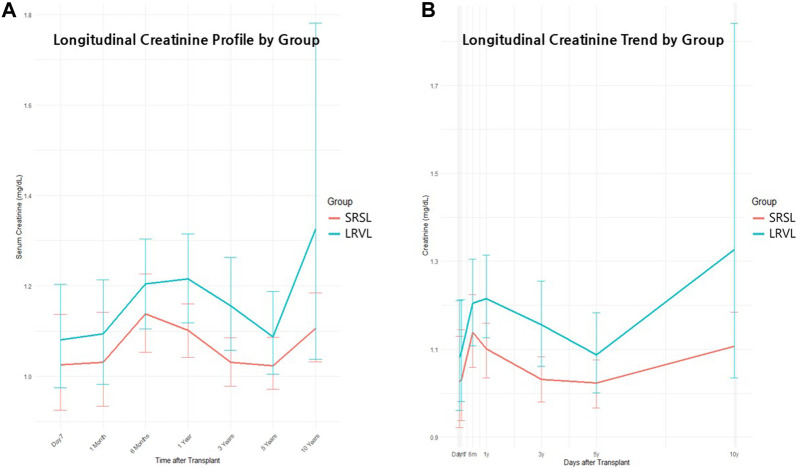
Longitudinal changes in serum creatinine after living donor liver transplantation. **(A)** Longitudinal creatinine profile by group. Mean serum creatinine levels with standard deviations after 1 week, 1 month, 6 months, 1 year, 3 years, 5 years, and 10 years are shown. The left renal vein ligation (LRVL) group (blue) demonstrated consistently higher creatinine values compared to the splenorenal shunt ligation (SRSL) group (red) across most time points. **(B)** Longitudinal trends for creatinine levels in each group. Creatinine trajectories during the same follow-up interval illustrate overall trends in both groups. The LRVL group exhibited a wider range of variability and relatively higher creatinine levels during long-term follow-up, with greater divergence observed at later time points.

Serum creatinine was analyzed at seven time points: After 1 week, 1 month, 6 months, 1 year, 3 years, 5 years, and 10 years. Both groups maintained generally stable renal function, with mean creatinine values near 1.2 mg/dL across the 10-year follow-up period (Figures 4A,B). However, the LRVL group demonstrated consistently higher mean creatinine levels at most time points. At year 10, mean creatinine was 1.11 ± 0.26 mg/dL in the SRSL group and 1.33 ± 1.57 mg/dL in the LRVL group. The large standard deviation in the LRVL group resulted from occasional extreme elevations, including one outlier with creatinine levels as high as 12.0 mg/dL.

Overall, renal function remained stable in most patients, but the LRVL group exhibited a broader range of creatinine variability that included occasional extreme elevations along with a gradual upward trend during long-term follow-up.

#### Fixed-Effects Modeling

To characterize temporal patterns, the follow-up period was divided into an early (0–6 months), an intermediate (1–5 years), and a late (5–10 years) phase. Overall, serum creatinine increased significantly with time (estimate +0.25, p = 0.018).

Phase-specific changes revealed that baseline creatinine at the start of the intermediate phase was significantly higher than in the early phase (estimate +0.153, p = 0.032), although the subsequent rate of increase slowed down (Time × Phase 1–5 years: −0.283, p = 0.010). A similar pattern was observed in the late phase (−0.216, p = 0.043).

LRVL group assignment was not independently associated with a statistically significant elevation in creatinine (estimate +0.092, p = 0.185). Nonetheless, the LRVL group showed a consistent longitudinal trend toward higher creatinine values, although this did not reach statistical significance (Table 4).

**TABLE 4:** Fixed-effects analysis of serum creatinine changes over time and by subgroup (SRSL vs. LRVL).

Term	Estimate	Std. Error	df	t value	p-value	Interpretation
Intercept	1.000	0.056	182.88	17.97	<0.001	Baseline Cr at time 0 in the reference phase
Time (per year)	0.250	0.106	644.87	2.37	0.018	Cr increases over time in reference phase
Phase: Year 1–5	0.153	0.071	644.17	2.16	0.032	Higher baseline Cr between intermediate phase vs. early phase
Phase: Year ≥5	−0.151	0.098	643.20	−1.54	0.123	Lower baseline Cr in 5+ years phase (not significant)
Group (LRVL vs. SRSL)	0.092	0.069	110.37	1.33	0.185	Slightly higher Cr in LRVL group (not significant)
Time × phase (Year 1–5)	−0.283	0.110	644.61	−2.58	0.010	Cr rises slower over time between 1 and 5 years
Time × phase (Year ≥5)	−0.216	0.106	644.82	−2.03	0.043	Slower Cr increase in late phase

Cr, serum creatinine; SRSL, splenorenal shunt ligation; LRVL, left renal vein ligation; y, year; df, degrees of freedom; Std., standard.

#### Linear Mixed-Effects Model for Changes in Creatinine

Changes in creatinine (ΔCr) were compared across the three defined phases. Although intergroup differences were not statistically significant in any of the phases, the pattern for each of the phases differed (Table 5): During the early phase, the value for the SRSL group was +0.098 mg/dL and that for the LRVL group was +0.147 mg/dL. The increase in the LRVL group approached significance (95% CI: −0.011 to +0.306). During the intermediate phase, both groups showed mild declines (SRSL: −0.080 mg/dL; LRVL: −0.125 mg/dL). Finally, only the LRVL group demonstrated a significant increase in the late phase (+0.246 mg/dL; 95% CI: +0.082 to +0.411, p < 0.05), whereas the SRSL group did not (+0.087 mg/dL, NS).

**TABLE 5:** Estimated change in serum creatinine level (ΔCr) across follow-up intervals according to group (SRSL vs. LRVL).

Phase	Group	Estimated ΔCr (mg/dL)	Std. Error	95% CI	Interpretation
Early 0 → 1 year	SRSL	0.0983	0.0827	[−0.0645, +0.2611]	Slight increase (not significant)
	LRVL	0.1473	0.0805	[−0.0111, +0.3057]	Slight increase, marginally close to significance
Intermediate 1 → 5 years	SRSL	−0.0800	0.0844	[−0.2460, +0.0860]	Mild decrease (not significant)
	LRVL	−0.1250	0.0828	[−0.2878, +0.0379]	Slightly larger decrease, not significant
Late 5 → 10 years	SRSL	0.0865	0.0870	[−0.0847, +0.2576]	Small increase (not significant)
	LRVL	0.2463	0.0836	[+0.0819, +0.4108]	Significant increase

Cr, serum creatinine; SRSL, splenorenal shunt ligation; LRVL, left renal vein ligation; CI, confidence interval; Std., standard; ΔCr, change in serum creatinine.

Despite the absence of statistically significant intergroup interactions, the pronounced late-phase increase in the LRVL group suggests a trend toward accumulating renal burden during extended follow-up.

### Longitudinal Trends in Tacrolimus Trough Levels

Tacrolimus trough levels decreased steadily over time in both groups, reflecting routine clinical tapering practices.

#### Overall Trends in Tacrolimus Trough Levels Over Time

**FIGURE 5:**
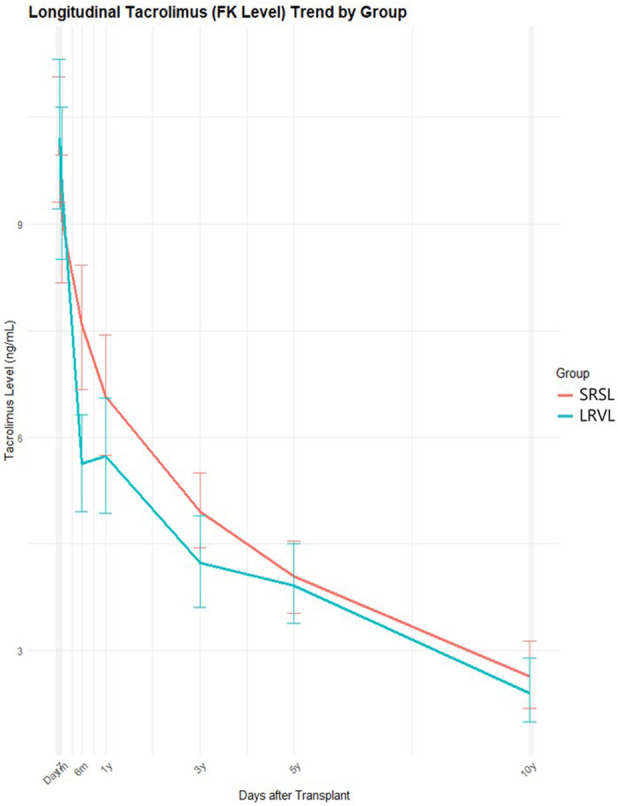
Longitudinal changes in tacrolimus levels in blood after transplantation. Tacrolimus levels were analyzed at seven fixed time points: at 1 week, 1 month, 6 months and 1, 3, 5, and 10 years. The left renal vein ligation (LRVL) group showed a more rapid decline in tacrolimus levels during the early phase compared to the splenorenal shunt ligation (SRSL) group, followed by a similar tapering pattern of immunosuppression in both groups. The early decrease in tacrolimus levels is presumed to be due to a rise in serum creatinine during the immediate postoperative period.

The LRVL group generally exhibited slightly lower tacrolimus levels than the SRSL group at all time points, with the most noticeable difference occurring during the early postoperative period (Figure 5).

#### Phase-specific Modeling

The fixed-effects model showed that tacrolimus levels declined rapidly in the early phase (p < 0.001), with a significantly faster rate of decrease in the LRVL group (interaction estimate: −4.820, p = 0.004). Levels continued to decrease during the intermediate phase (p < 0.001), but group differences were no longer significant. A mild further decline was observed in both groups during the late phase (p < 0.001), with no intergroup differences. These findings suggest that early tacrolimus tapering may have been more aggressive in the LRVL group, possibly reflecting concerns for renal function by the attending clinicians, while tapering strategies later converged for both groups. Overall, despite a tendency toward earlier tacrolimus tapering in the LRVL group, these patients demonstrated a more pronounced long-term decline in renal function, reflected by progressively higher creatinine levels and the exclusive occurrence of renal failure requiring dialysis treatment within this group during the late phase of the follow-up period (Table 6).

**TABLE 6:** Changes in tacrolimus trough levels over time by phase and group.

Phase	Variable	Estimate	Std. Error	p-value	Interpretation
Early 0 → 1 year	Intercept	9.875	0.395	<0.001	FK level at time = SRSL group is higher
	Time_year	−4.709	1.196	0.000	FK level significantly declines over time
	Group (LRVL vs. SRSL)	0.481	0.558	0.390	LRVL group shows slightly higher FK level (not significant)
	Time × group	−4.820	1.691	0.004	FK level in LRVL group declines faster than in SRSL group
Intermediate 1 → 5 years	Intercept	7.411	0.512	<0.001	FK level starts lower than in early phase
	Time_year	−0.832	0.204	0.000	FK level continues to decrease over time
	Group (LRVL vs. SRSL)	−0.915	0.714	0.202	LRVL group shows slightly lower FK baseline level (not significant)
	Time × group	0.069	0.284	0.807	No significant group difference in slope
Late 5 → 10 years	Intercept	5.501	0.468	<0.001	FK level further reduced
	Time_year	−0.291	0.055	<0.001	Decline continues at a slower rate
	Group (LRVL vs. SRSL)	−0.055	0.654	0.933	No baseline difference between groups
	Time × group	−0.016	0.077	0.839	No group difference in slope (not significant)

FK, level, Tacrolimus trough level (FK506); SRSL, splenorenal shunt ligation; LRVL, left renal vein ligation; NS, not significant.

## Discussion

Effective management of large spontaneous SRSs during LDLT is essential to ensure adequate portal inflow and prevent portal steal syndrome. Two intraoperative techniques are currently employed for this purpose: SRSL, which anatomically targets the shunt itself, and LRVL, which functionally interrupts shunt outflow by occluding the LRV. SRSL provides anatomically precise control but can be technically demanding in patients with dense adhesions or distorted venous anatomy. In contrast, LRVL is less technically challenging and is often preferred in hemodynamically unstable or surgically complex cases [1–3].

In this study, both techniques were effective in preventing portal steal and maintaining adequate graft perfusion. Patient and graft survival, re-transplantation rates, and shunt recanalization were comparable between groups, supporting LRVL as a feasible and effective alternative when direct shunt ligation is difficult or deemed unsafe.

Nevertheless, concerns remain regarding the potential long-term renal consequences of LRVL. Because the LRV is the primary venous drainage route of the left kidney, its ligation may predispose patients to impaired renal perfusion and progressive renal dysfunction, particularly in LDLT recipients who require lifelong exposure to nephrotoxic immunosuppressants such as tacrolimus. Although previous nontransplant studies have suggested minimal long-term renal impact after LRV ligation, these findings may not be fully applicable to liver transplant recipients under chronic immunosuppression. Beyond simple mechanical obstruction of venous outflow, progressive renal dysfunction after LRVL may involve multiple pathophysiological mechanisms, including microvascular injury, altered renal autoregulation, and synergistic nephrotoxicity associated with prolonged calcineurin inhibitor exposure. These mechanisms may partly explain the greater variability in creatinine levels and the exclusive occurrence of dialysis in the LRVL group during long-term follow-up.

To evaluate renal outcomes more rigorously, we performed 1:1 propensity score matching using the baseline values for relevant variables. After matching, long-term tacrolimus exposure was comparable between the groups, although the LRVL group demonstrated a tendency toward earlier tacrolimus tapering during the early postoperative period, likely reflecting concerns by the clinicians about renal function. Despite this early tapering, the LRVL group exhibited a sustained trend toward higher serum creatinine throughout follow-up, and all cases of renal failure requiring dialysis occurred exclusively in the LRVL groups between 5 and 10 years post-transplant. Although not definitive, these findings raise the possibility that LRVL may be associated with a subtle but progressive long-term renal burden.

These observations align with prior experience at our institution. In an early report, Lee et al. found no short-term deterioration in renal function, proteinuria, or hematuria following LRVL [4]. However, a 20-year follow-up study by Hwang et al. in the same patient cohort revealed that 9.1% of them eventually required dialysis, and more than half experienced a decline in the estimated glomerular filtration rate [12]. Although underlying comorbidities such as diabetes or glomerulonephritis likely contributed to this outcome, chronic venous congestion from LRV ligation may also have played a role in the progressive renal decline. These longer-term findings underscore the importance of careful patient selection and appropriate renal monitoring following LRVL.

Our study provides follow-up data up to 10 years after transplantation. While no statistically significant differences in renal failure were observed during this interval, the trends in creatinine elevation and the exclusive occurrence of cases requiring dialysis in the LRVL group suggest that more pronounced differences may emerge with a more extended follow-up, as suggested by the long-term data reported by a Hwang et al. [12] This highlights the need for continued surveillance and emphasizes the importance of choosing surgical strategies that minimize long-term renal risk.

In this context, the potential advantages of SRSL warrant renewed attention. SRSL preserves physiological renal venous drainage and avoids direct compromise of kidney perfusion. Although it may be technically challenging, particularly in the presence of dense adhesions, it provides targeted shunt interruption while minimizing collateral vessel injury. By avoiding alterations in systemic venous return, SRSL may offer the advantage of sparing the kidney, which is particularly important in patients with pre-existing renal risk factors.

At our institution, SRSL has consistently provided reliable portal decompression and stable long-term renal outcomes. Given the rising prevalence of chronic kidney disease after liver transplantation and the widespread use of tacrolimus, SRSL may represent the preferred SRS management strategy when anatomically feasible. Surgical planning should incorporate both anatomical considerations and long-term organ protection, particularly in recipients predisposed to renal dysfunction.

This study has several strengths, including a relatively large cohort of LDLT recipients with large SRS, long-term follow-up of up to 10 years, and rigorous propensity score matching to minimize selection bias. In addition, standardized immunosuppressive protocols and objective renal biomarkers further strengthened the validity of the comparative analyses. However, several limitations should be acknowledged. This was a retrospective single-center study, which limits generalizability, and residual confounding cannot be completely excluded. Procedural selection was influenced by intraoperative assessment, surgeon preference, and individual judgment, introducing potential selection bias. Although we attempted to mitigate this by using MELD score and RBC transfusion as surrogate markers in the propensity score model, unmeasured surgeon-related factors may still have contributed to residual confounding. Furthermore, technical difficulty in determining the ligation strategy was difficult to quantify objectively, and renal function was primarily assessed using only serum creatinine. and the limited number of patients requiring dialysis reduced the statistical power for detecting differences in rare outcomes. Another limitation is that detailed graft function parameters, including rejection and longitudinal liver function tests, were not systematically analyzed. Therefore, subtle differences in graft function between groups may not have been fully captured. These strengths and limitations should be considered when interpreting our findings, and further prospective multicenter studies are warranted to validate our results.

In conclusion, both SRSL and LRVL are effective strategies for managing SRS during LDLT. Although survival outcomes were comparable, LRVL may be associated with potential long-term renal risks, even when early tacrolimus tapering is implemented. When anatomically feasible, SRSL may be considered, whereas LRVL remains an important alternative in technically challenging or hemodynamically unstable cases. In patients requiring LRVL, strategies aimed at renal protection, including careful titration of nephrotoxic immunosuppressants, should be implemented, and long-term renal function should be closely monitored. Further prospective multicenter studies with extended follow-up are warranted to validate these findings and guide optimal surgical decision-making in the management of SRS during liver transplantation.

## Data Availability

The data analyzed in this study is subject to the following licenses/restrictions: The dataset analyzed during this study contains sensitive patient medical information obtained from a single tertiary transplant center. Due to institutional and ethical restrictions, the raw data cannot be made publicly available. Access to the dataset may be granted upon reasonable request to the corresponding author and subject to approval by the Institutional Review Board and data-sharing agreements in compliance with patient privacy protection regulations. Requests to access these datasets should be directed to romikwh@gmail.com.

## References

[1] Moon DB Lee SG Kim KH Ahn CS Hwang S Park KM The Significance of Complete Interruption of Large Spontaneous Portosystemic Collaterals in Adult Living Donor Liver Transplantation as a Graft Salvage Procedure. Transpl Int (2008) 21(7):698–700. 10.1111/j.1432-2277.2008.00639.x 18384468

[2] Ikegami T Shirabe K Nakagawara H Yoshizumi T Toshima T Soejima Y Obstructing Spontaneous Major Shunt Vessels Is Mandatory to Keep Adequate Portal Inflow in Living-Donor Liver Transplantation. Transplantation (2013) 95(10):1270–7. 10.1097/TP.0b013e318288cadc 23598942

[3] Sadamori H Yagi T Matsukawa H Matsuda H Shinoura S Umeda Y The Outcome of Living Donor Liver Transplantation with Prior Spontaneous Large Portasystemic Shunts. Transpl Int (2008) 21(2):156–62. 10.1111/j.1432-2277.2007.00593.x 18005086

[4] Lee SG Moon DB Ahn CS Kim KH Hwang S Park KM Ligation of Left Renal Vein for Large Spontaneous Splenorenal Shunt to Prevent Portal Flow Steal in Adult Living Donor Liver Transplantation. Transpl Int (2007) 20(1):45–50. 10.1111/j.1432-2277.2006.00392.x 17181652

[5] Kang WH Moon DB Ko GY Gwon DI Yoon YI Cho HD Application of Proximal Splenic Vein Embolization to Interrupt Complicated Large Splenorenal Shunts in Adult Living Donor Liver Transplantation. Ann Surg (2022) 276(6):e834–e41. 10.1097/SLA.0000000000004868 33914461

[6] Loveday BPT Dib MJ Sequeira S Alotaiby N Visser R Barbas AS Renal Outcomes Following Left Renal Vein Harvest for Venous Reconstruction During Pancreas and Liver Surgery. HPB (Oxford) (2019) 21(1):114–20. 10.1016/j.hpb.2018.07.015 30322713

[7] Ojo AO Held PJ Port FK Wolfe RA Leichtman AB Young EW Chronic Renal Failure After Transplantation of a Nonrenal Organ. N Engl J Med (2003) 349(10):931–40. 10.1056/NEJMoa021744 12954741

[8] Samson RH Lepore MR Jr. Showalter DP Nair DG Lanoue JB . Long-Term Safety of Left Renal Vein Division and Ligation to Expedite Complex Abdominal Aortic Surgery. J Vasc Surg (2009) 50(3):500–4. ; discussion 4. 10.1016/j.jvs.2009.04.041 19595540

[9] Pandirajan K Katsogridakis E Sidloff D Sayers RD Bown MJ Saratzis A . Effects of Left Renal Vein Ligation During Open Abdominal Aortic Aneurysm Repair on Renal Function. Eur J Vasc Endovasc Surg (2020) 60(6):829–35. 10.1016/j.ejvs.2020.08.003 32912760

[10] Moon DB Lee SG Ahn C Hwang S Kim KH Ha T Application of Intraoperative Cine-Portogram to Detect Spontaneous Portosystemic Collaterals Missed by Intraoperative Doppler Exam in Adult Living Donor Liver Transplantation. Liver Transpl (2007) 13(9):1279–84. 10.1002/lt.21252 17763379

[11] Lim SY Wang R Tan DJH Ng CH Lim WH Quek J A Meta-Analysis of the Cumulative Incidence, Risk Factors, and Clinical Outcomes Associated with Chronic Kidney Disease After Liver Transplantation. Transpl Int (2021) 34(12):2524–33. 10.1111/tri.14149 34714569

[12] Ko HJ Hwang S Kang J Moon DB Ahn CS Ha TY Long-Term Outcomes of Left Renal Vein Ligation in Living Donor Liver Transplantation: A 20-Year Study. Ann Transpl (2025) 30:e947492. 10.12659/AOT.947492 40195082 PMC11992949

